# Polymer Electrode Materials for Sodium-ion Batteries

**DOI:** 10.3390/ma11122567

**Published:** 2018-12-17

**Authors:** Qinglan Zhao, Andrew K. Whittaker, X. S. Zhao

**Affiliations:** 1 School of Chemical Engineering, The University of Queensland, Brisbane 4072, Australia; qinglanangela.zhao@uq.edu.au; 2 Australian Institute for Bioengineering and Nanotechnology and ARC Centre of Excellence in Convergent Bio-Nano Science and Technology, The University of Queensland, Brisbane 4072, Australia; a.whittaker@uq.edu.au

**Keywords:** polymer, organic electrode material, sodium-ion battery

## Abstract

Sodium-ion batteries are promising alternative electrochemical energy storage devices due to the abundance of sodium resources. One of the challenges currently hindering the development of the sodium-ion battery technology is the lack of electrode materials suitable for reversibly storing/releasing sodium ions for a sufficiently long lifetime. Redox-active polymers provide opportunities for developing advanced electrode materials for sodium-ion batteries because of their structural diversity and flexibility, surface functionalities and tenability, and low cost. This review provides a short yet concise summary of recent developments in polymer electrode materials for sodium-ion batteries. Challenges facing polymer electrode materials for sodium-ion batteries are identified and analyzed. Strategies for improving polymer electrochemical performance are discussed. Future research perspectives in this important field are projected.

## 1. Introduction

### 1.1. The Importance of Energy Storage

The ever growing and increasing demand for energy and the related environmental issues have become two major challenges to human society in the 21st century [1]. In order to address these challenges, worldwide effort has been invested into making full use of renewable and sustainable energy resources, such as wind and solar energy. However, these energy resources are intermittent and supply is not predictable, thus requiring systems to store the energy collected [2]. With the massive growth of renewable energy sources, energy storage is becoming more and more important and indispensable to the development of renewable energy.

### 1.2. Sodium-Ion Batteries

Lithium-ion batteries (LIBs) currently dominate the energy storage market [3]. However, lithium resources are geographically limited [4], and the relative abundance in the Earth’s crust is only 20–65 ppm [5]. The increasingly growing demand for lithium will ultimately lead to dramatically increased prices, potentially making lithium the “new gold” [6]. Thus, it is urgent to develop alternative battery technologies to move away from lithium [7].

Sodium-ion batteries (NIBs) are considered the most promising alternative to LIBs due to the lower cost of the metal resource [8,9]. NIBs were initially investigated together with LIBs from the 1970s to the 1980s [10,11,12]. After this initial activity, development of NIBs stalled in the 1990s due to the commercial success of LIBs with carbonaceous material as the anode and lithium cobalt oxide as the cathode [13]. The current anode material used in LIBs is graphite. Unfortunately, graphite has little capacity for storing sodium ions due to the larger ionic radius of sodium compared with lithium [14]. In 2000, Stevens and Dahe [15] reported that hard carbon (e.g., non-graphitisable carbon) exhibits a reversible sodium-ion storage capacity of about 300 mAh·g^−1^, comparable to graphitic carbon for lithium ion storage. This research, along with the above-mentioned challenges for LIBs, has led to a resurgence in research and development of NIBs [16,17,18].

### 1.3. Electrode Materials for Sodium-Ion Batteries

Current electrode materials for NIBs include inorganic compounds (e.g., transition metal oxides, phosphates, fluorides, sulphides, phosphides, alloys, Prussian blue analogues and carbonaceous materials) [18,19,20,21,22,23] and organic materials [24,25]. Many inorganic compounds contain toxic metals, causing further concerns over resources and environmental contamination. In addition, the inorganic compounds undergo the insertion-type sodium storage mechanism. During the intercalation and deintercalation of sodium ions (1.02 Å), large volume changes and irreversible phase transitions may occur, leading to low reversible capacity and poor cycling performance [26].

Redox-active organic materials are gaining increasing research interest due to a number of intrinsic advantages [27]. First, organic materials contain no heavy metals, an increasingly important consideration for development of green energy storage devices [28]. Second, the cost can be reduced because organic materials can be produced from renewable natural resources [29]. Third, a range of polymer structures with different surface functionalities can be designed at the molecular level [30]. Fourth, organic materials exploit an alkali ion storage involving reactions of chemical bonds, making it less sensitive to the large sodium ion [31]. Finally, organic materials are inherently flexible, making it possible to realize commercial flexible energy storage devices [32].

### 1.4. Polymer Electrode Materials for Sodium-Ion Batteries

Organic electrode materials can include small organic molecules and polymers. Most small organic compounds suffer from rapid dissolution into organic electrolyte, leading to a short cycle life. Polymerization is considered a simple and efficient way to mitigate this problem [33]. Compared with small organic molecules, polymers with large extended backbones of repeating units have lower if not negligible solubility in electrolytes and thus better cycling performance. The advantages and disadvantages of electrode materials for sodium-ion batteries are listed in Table 1.

The reported polymer electrode materials for NIBs can be mainly divided into three categories according to the redox reactions they undergo. The first involves reactions at the C=O groups, and includes the polyimides and polyquinones. The second is based on reactions of C=N groups, and the typical representatives are the Schiff base polymers. The third type encompasses conducting polymers exploiting doping reactions, such as conjugated conductive polymers and non-conjugated conductive radical-containing polymers. However, apart from conducting polymers, most of the polymers have poor conductivity, and this remains one of the main challenges to wider exploitation of polymeric electrode materials.

This review provides a summary of recent research and developments of polymer electrode materials for NIBs. The evolution of polymeric electrode materials from the initial work on polyimides, their electrochemical performance and sodium storage mechanisms will be discussed. The current grand challenges and strategies for improving polymer electrode materials for NIBs are identified and analyzed.

## 2. Polymer Electrode Materials for NIBs

### 2.1. Polymers with Carbonyl Functional Groups

#### 2.1.1. Polyimides

In the classical approach, aromatic polyimides are synthesized in two steps [34]. Initially, a dianhydride and a diamine are reacted in a polar aprotic solvent to generate a polyamic acid. In the second step, heating or treatment with chemical dehydrating agents is required to cyclodehydrate the polyamic acid to form the polyimide. The polyimide can be also obtained by a one-step solvothermal synthesis, in which the polycondensation and imidization occur spontaneously [34,35].

**Figure 1:**
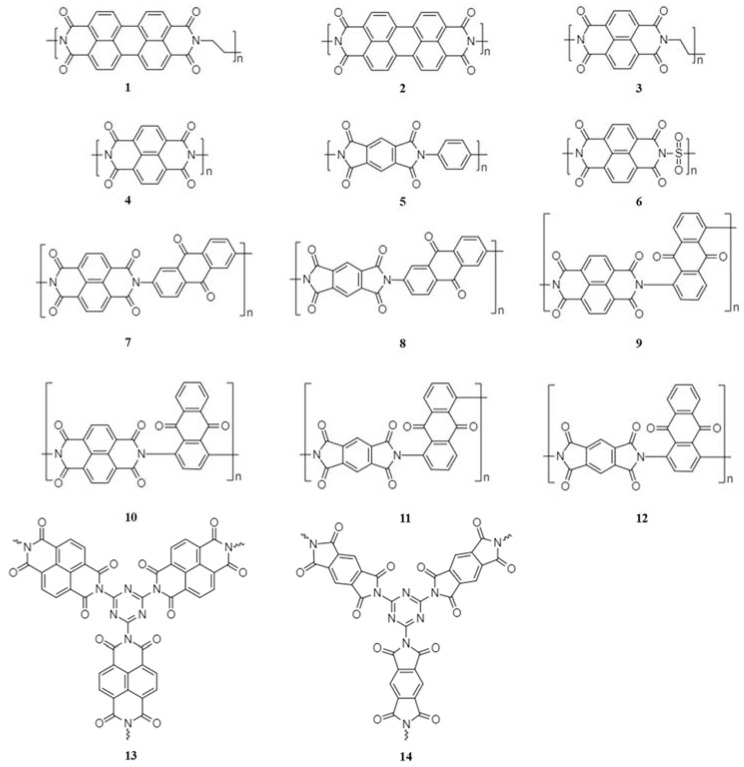
The structure of typical polyimides suitable for use in NIBs.

The structure of the dianhydride and diamine precursors determines the nature of the aromatic backbone of the polyimides. Therefore, various molecular structures of polyimides (Figure 1) can be realized through judicious choice of the dianhydrides and diamines used in the synthesis.

**Scheme 1:**
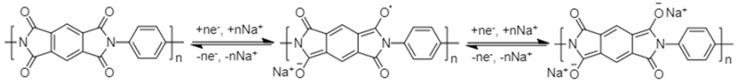
Schematic representation of the redox reaction of aromatic polyimides.

Aromatic polyimides are redox-active polymers (Figure 1), and hence are very promising energy storage electrode materials [36]. The carbonyls of the polyimide provide the active sites for the redox reaction. The carbonyls can interact with sodium ions via enolization, involving two one-electron reduction steps to yield sequentially the anion radical and the dianion (Scheme 1).

The polyimides currently used in NIBs are usually derived from 3,4,9,10- perylenetetracarboxylic dianhydride (PTCDA) (**1**, **2**) [37,38], 1,4,5,8-naphthalenetetracarboxylic dianhydride (NTCDA) (**3**, **4**) [39,40] and pyromellitic dianhydride (PMDA) (**5**) [41]. The average discharge voltage of polyimides becomes progressively lower when the aromatic core changes from PTCDA (1.94 V) to NTCDA (1.89 V) and to PMDA (1.73 V) [37]. This is due to the increased energy of the lowest unoccupied molecular orbital (LUMO) and thus the decreased average discharge voltage. Therefore, PTCDA-derived polyimides are most commonly investigated as cathode materials for NIBs [37,38], while the NTCDA- and PMDA-derived polyimides are studied as anode materials [39,40,41]. Improved cycling stability and increased gravimetric capacity can be achieved by employing larger conjugated aromatic cores and reducing the molecule weight of inactive chains, respectively.

The working voltage of the polyimides can be manipulated by introducing an electron withdrawing group. For example, compared with the polyimide containing an ethylene connecting unit, polyimide (**6**) with a connecting sulfonyl group has a 0.14 V higher charge-discharge voltage, resulting from the decreased electron density of the redox-active carbonyl by inductive effects [42].

A very attractive advantage of polyimides as electrode materials for organic NIBs is their insolubility in electrolytes, which results in good cycling stability. However, only half of the carbonyls can be involved in the redox reactions and so the capacity of polyimides is usually less than 140 mAh·g^−1^ (Table 2). Further development of polyimide electrode materials requires this bottleneck to be overcome. Copolymerization of the imide with a high-capacity quinone is a promising strategy to obtain polyimides with enhanced capacity. Xu et al. prepared two imide-quinone copolymers with high reversible capacities of 192 mAh·g^−1^ (**7**) and 165 (**8**) mAh·g^−1^ [43]. A number of other imide-quinone copolymers (**9**, **10**, **11**, **12**) have also displayed improved capacities [44]. In addition, introduction of electron withdrawing groups into the polyimide chain can increase the capacity of the polyimides. Li et al. reported that the introduction of the triazine ring can increase the sodium-ion storage capability (**13**, **14**), through the synergistic effects of electron withdrawing amide groups and triazine rings [45].

#### 2.1.2. Polyquinones

The polyquinones employed as electrode materials for NIBs are usually sulfur-containing polyquinones, obtained by the polycondensation reaction of quinone monomers and sulfides [46,47,48,49]. Due to the reductive capacity of the quinonyl groups, a side redox reaction between the quinonyl groups and sulfide anions may occur during the formation of the polyquinone main chains. Therefore, the synthesis is usually followed by subsequent purification or oxidation steps [47,49].

**Figure 2:**
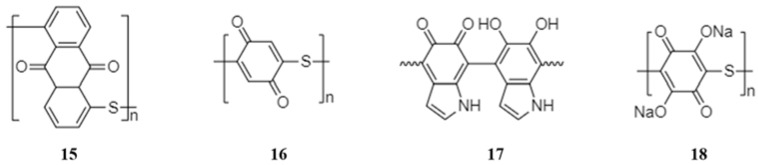
The structure of several polyquinones suitable for NIBs.

As is shown in Figure 2, the structure of the polyquinone chains is determined by the molecular structure of quinone monomers. The polyquinones usually contain a chain of quinone rings separated by heteroatoms with an unshared electron pair. Though the heteroatoms are not the active sites and do not contribute to the sodium storage, the heteroatoms can enhance the discharge voltage [48].

**Scheme 2:**
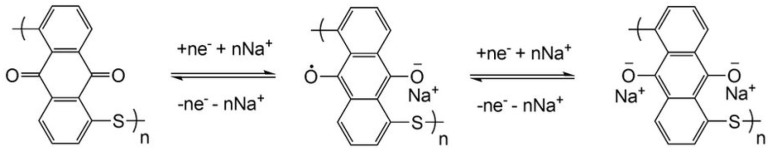
Schematic representation of the redox reaction of polyquinones.

Similar to polyimides, polyquinones usually undergo two-electron redox reactions through reversible electrochemical association of sodium ions with its redox-active quinonyl groups (Scheme 2).

**Figure 3:**
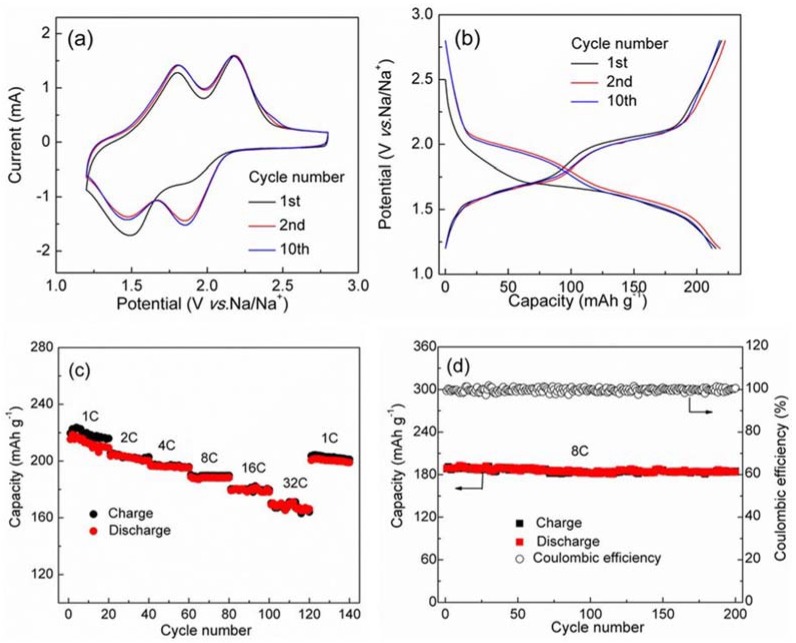
Electrochemical performance of polymer **15** as candidate anode material for sodium-ion batteries: (**a**) cyclic voltammograms (CV) curves at a scan rate of 5 mV·s^−1^; (**b**) charge and discharge profiles at a current density of 180 mA·g^−1^; (**c**) rate performance (1C = 200 mA·g^−1^); (**d**) cycling performance at 8 C rate (1600 mA·g^−1^). Adapted from [46], with permission from © 2013 Springer Nature.

The first polyquinone used in NIBs was poly(anthraquinonyl sulfide) (**15**), reported by Yang and co-workers in 2013 [46]. The poly(anthraquinonyl sulfide) displayed two pairs of symmetric redox peaks at 1.85/2.18 V and at 1.46/1.8 V (Figure 3a), implying a two-step redox reaction of the anthraquinonyl groups. Consistent with this, the material displayed two plateaus at 1.5 and 2.0 V with a capacity of 220 mAh·g^−1^ (Figure 3b), corresponding to 98% theoretical capacity of storage of two sodium ions. The polyquinone showed a good rate capability with the capacity of 160 mAh·g^−1^ at a high current density of 6400 mA·g^−1^ and a stable cyclability with 85% capacity retention at the 500th cycle at 1600 mA·g^−1^ (Figure 3c,d).

In 2015, poly(benzoquinonyl sulfide) (**16**) was reported by Zhou and co-workers [47]. This polymer was synthesized by an oxidative polymerization technique due to the high reactivity of the benzoquinone monomers. The poly(benzoquinonyl sulfide) delivered a high capacity of 268 mAh·g^−1^ and a high energy density of 557 Wh·kg^−1^. However, the capacity decayed at a rate of 0.88 mAh·g^−1^·cycle^−1^ after the 2nd cycle and remained at only 68 % after 100 cycles. The capacity was also unstable at current densities above 200 mA·g^−1^. The authors ascribed the unsatisfactory cycling stability and rate performance to the poor compatibility between the sodium and the 1 mol·L^−1^ NaN(CF_3_SO_2_)_2_/(dioxolane + dimethoxy ethane) electrolyte.

Besides the above synthetic polymers, biomass-derived polydopamine (**17**) is an ideal redox-active biodegradable electrode material. The carbonyl groups of the *o*-benzoquinone group act as the active sites for coordination of sodium ions. The polydopamine exhibited excellent electrochemical performance in NIBs, with a high capacity of 500 mAh·g^−1^ and a capacity retention of 100 % at the 1024th cycle at a current density of 50 mA·g^−1^ [48].

**Figure 4:**
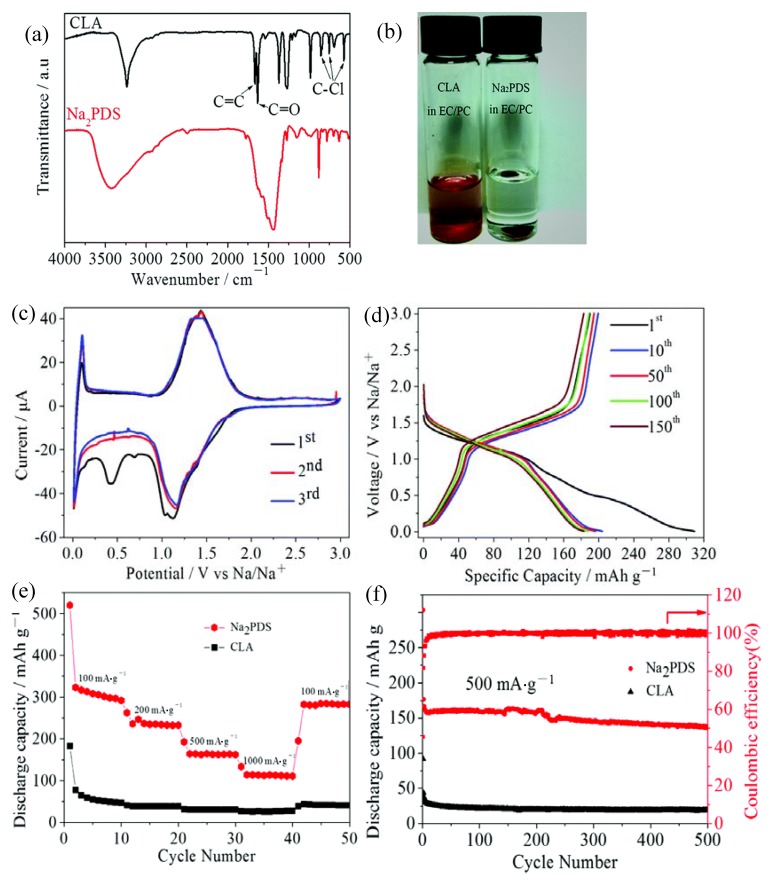
(**a**) Fourier transform infrared spectra of 2,5-dichloro-3,6-dihydroxy-*p*-quinone (CLA) and sodium salt of poly(2,5-dihydroxy-*p*-benzoquinonyl sulfide) (Na_2_PDS); (**b**) photograph of the CLA powder and the Na_2_PDS electrode in the electrolyte; (**c**) CV curves of the Na_2_PDS electrode at a scan rate of 0.1 mV·s^−1^; (**d**) discharge and charge profiles of the Na_2_PDS electrode at a current density of 100 mA·g^−1^; (**e**) rate performance and (**f**) cycling performance of CLA and Na_2_PDS as candidate anode materials for sodium-ion batteries. Adapted from [50], with permission from © 1996 Royal Society of Chemistry.

The emergence of the polyquinones resulted from the high tendency for dissolution of quinones in the organic electrolytes [49]. However, polymerization can only mitigate rather than totally prevent the dissolution. Wu et al. [50] reported a sodium salt of poly(2,5-dihydroxy-*p*-benzoquinonyl sulfide) (**18**) as a novel anode material for NIBs. As is seen from Figure 4, the intrinsically insoluble salt form of the polyquinone exhibited an excellent cyclability with a capacity of about 138 mAh·g^−1^ at the 500th cycle, much better than the original quinone (19 mAh·g^−1^). Thus, formation of the salt can be a feasible solution to the problem of solubilisation, although further efforts are required in this direction.

### 2.2. Schiff Base Polymer Electrode Materials

#### 2.2.1. Synthesis and Structural Diversity

**Figure 5:**
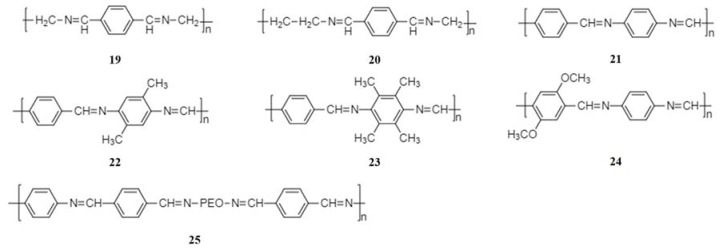
Structure of Schiff base polymers.

Schiff base polymers can be synthesized by a simple polycondensation reaction of diamines and dialdehydes or diketones [51]. Schiff base polymers contain a system of conjugated –C=C– and –C=N– bonds. The structure of diamine and dialdehyde or diketone precursors determines the diversity of the Schiff base polymers (Figure 5).

#### 2.2.2. Charge Storage Mechanism in Schiff Base Polymers

**Scheme 3:**
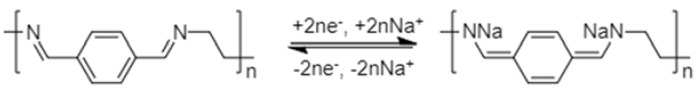
Schematic representation of sodium-ion storage in Schiff base polymers.

Schiff base polymers are increasingly being explored as organic electrode materials for NIBs. The active centre of Schiff base polymers for the sodium-ion storage is the C=N of conjugated –N=CH–Ar–CH=N– (Ar = aromatic ring) (Scheme 3). Planarity and conjugation are the key properties required for electrochemical activity of Schiff base polymers [52]. The “reverse” configuration –C=NH–Ar–NH=C–, though isoelectronic, is not electrochemically active.

#### 2.2.3. Recent Development of Schiff Base Polymer Electrode Materials for NIBs

The first study on Schiff base polymers (**19**–**24**) as anode materials for NIBs was reported by the group of Armand in 2014 [52]. It was found that the reduction reaction takes place at potentials below 1.5 V vs. Na/Na^+^, indicating that Schiff base polymers are very promising anode materials for NIBs. The redox potential can be adjusted by addition of appropriate substituents without compromising the polymer chain planarity and extent of conjugation. A maximum capacity of 350 mAh·g^−1^ was achieved on addition of 50 wt% of Ketjen Black, corresponding to 2.8 sodium ions per monomer unit.

Recently, the Armand group reported a polySchiff-polyether terpolymer (**25**) with an electrochemical activity at low redox potential vs. Na/Na^+^ and self-binding property, allowing the preparation of laminated electrodes without binder [53]. At the same carbon content, the laminate electrode delivered higher capacities than the powder counterpart due to superior cohesive inter-particle contact. A stable reversible capacity of ~185 mAh·g^−1^ could be obtained for the binder-free laminated electrode. Due to its intriguing adhesive properties, this polySchiff-polyether terpolymer was also tested as a binder for hard carbon, representing a proof-of-concept in the field of redox-active binders.

#### 2.2.4. Challenges and Opportunities in Developing Schiff Base Polymer Electrode Materials

The low solubility of Schiff base polymers in most organic solvents [54] is an advantage for electrode materials; however, this makes the materials difficult to process. The balance between the solubility and processability can be manipulated by modifying the polymer chain using appropriate linkers between the azomethine units [52,53,54]. Furthermore, the electrical conductivity of Schiff base polymers can be increased up to the level of semiconductors by doping with iodine [55]. This important property makes Schiff base polymers very promising electrode materials for NIBs.

### 2.3. Conducting Polymer Electrode Materials

#### 2.3.1. Conjugated Conducting Polymers

**Scheme 4:**
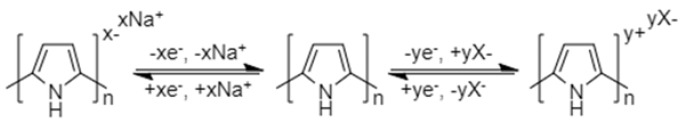
Schematic representation of p-doping and/or n-doping reactions of conjugated conductive polymers.

**Figure 6:**
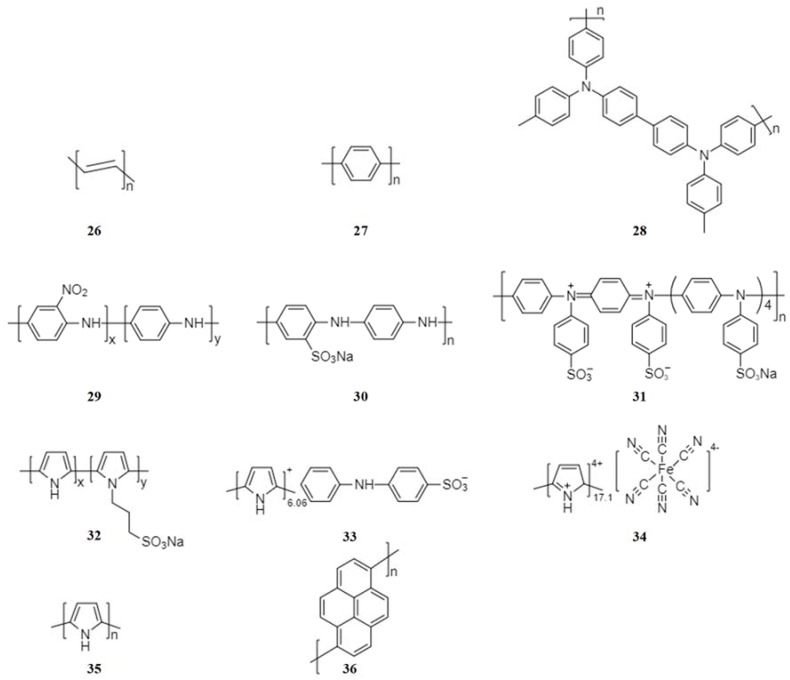
The structure of conjugated conductive polymers suitable for NIBs.

Conjugated conducting polymers (Figure 6) can undergo p-doping and/or n-doping reactions (Scheme 4). In p-doping reactions, the polymer changes to the positively charged state and reacts with anions such as PF_6^−^_. In n-doping, the polymer changes to the negatively charged state and reacts with the sodium cations.

The first study of conjugated conductive polymers for NIBs was reported by Shacklette et al. in 1985 [56]. It was found that polyacetylene (PAc) (**26**) and polyparaphenylene (PPP) (**27**) are possible anode materials. PAc and PPP are bipolar polymers, capable of undergoing both p-doping and n-doping redox reactions [27]. The bipolar polymers have potential in all-organic batteries since they can act as both cathode and anode.

Most of the well-studies conjugated conductive polymers are p-type polymers, such as polyaniline (PANi) and Polypyrrole (PPy). P-type conducting polymers (**28**, **29**) can be used in the cathode to construct all-organic batteries with n-doping polymers as the anode [46,57]. However, the p-doping mechanism can be modified by introducing electron withdrawing groups. For example, by grafting the electron withdrawing -SO_3_Na group onto the polyaniline chain, the sulfonated polyaniline/Ketjen black composite (**30**) mainly experienced an insertion/extraction mechanism of sodium ions rather than p-doping reactions [58]. A similar strategy was implemented for poly(diphenylaminesulfonic acid sodium) (**31**) [59] and poly(pyrrole-co-(sodium-3-(pyrrol-lyl) propanesulphonate)) (**32**) [60].

The advantage of conducting polymers over other polymers is their high electric conductivity comparable to semiconductors or even metals [61]. However, their capacity is low and limited by the degree of doping. Incorporation of a high-capacity redox-active unit can be used to overcome this drawback, as extensively studied by Yang’s group [62,63,64]. By grafting o-nitroaniline groups onto polyaniline chains, polymer (**29**) achieved a high reversible capacity of 180 mAh·g^−1^ and retained at 173 mAh·g^−1^ at the 50th cycle [62]. Similarly, by doping with diphenylamine sulfonate anions (**33**) [63] and ferrocyanides (**34**) [64], the anion-doped polypyrroles demonstrated increased capacities due to the contribution from the doped redox-active units. Thus, to develop high-performance conjugated conductive polymer electrode materials, it is feasible and promising to focus on the incorporation of other redox-active units onto the polymer chain.

**Figure 7:**
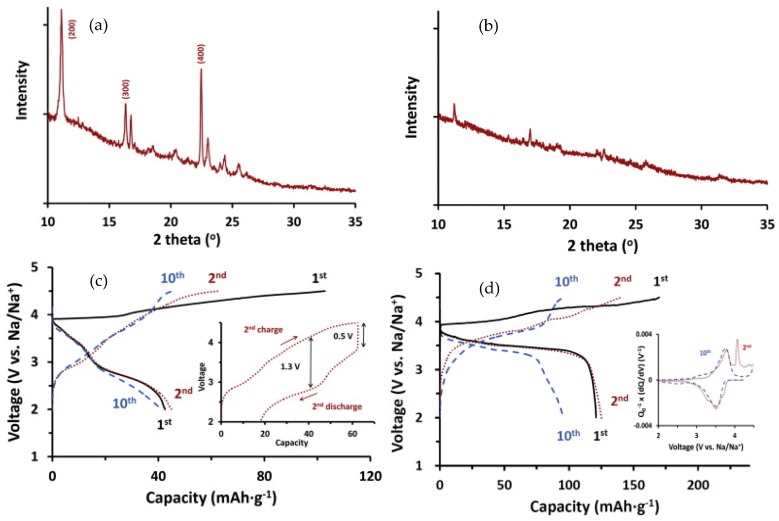
(**a**,**b**) XRD patterns and (**c**,d) charge and discharge profiles at a current density of 20 mA·g^−1^ of crystalline oligopyrene (**a**,**c**) and amorphous oligopyrene (**b**,**d**) as candidate cathode materials for sodium-ion batteries; Inset of (**c**) shows the 2nd charge and discharge profile to indicate a large overpotential; Inset of (**d**) shows the differential capacity (dQ/dV) curves for the 2nd and 10th cycles. Adapted from [68], with permission from © 2013 Elsevier.

It has been reported that the particle size, morphology, crystalline structure of conjugated conductive polymers may affect their electrochemical performance. Wang’s group [65] found that submicron polypyrrole (**35**) with fluffy structure and chain-like morphology showed a higher capacity (183 mAh·g^−1^) than bulk polypyrrole (34.8 mAh·g^−1^). The excellent electrochemical performance is due to the unique structure, which increased the electrical contact between the polypyrrole particles and promoted the penetration of the electrolyte into the material. In addition, hollow nanospheres [66] and mesoporous nanosheets [67] of polypyrrole were prepared through the use of soft templates. The unique morphology enabled stable cycling performance and superior rate capability of the polypyrrole electrodes. Han et al. [68] reported crystalline and amorphous oligopyrenes (**36**) as high-voltage cathodes for sodium-ion batteries. The crystalline oligopyrene with a layered structure (Figure 7a) exhibited sloping charge and discharge curves with a discharge capacity of 42.5 mAh·g^−1^ and an average potential of 2.9 V vs. Na/Na^+^ at a current density of 20 mA·g^−1^ (Figure 7c). The inset in Figure 7c shows a large overpotential, which was ascribed to the slow ClO_4_^−^ diffusion through the crystalline phase. The amorphous oligopyrene showed substantially reduced overpotential with a higher capacity of ~120 mAh·g^−1^ and a discharge plateau at 3.5 V vs. Na/Na^+^ (Figure 7d).

#### 2.3.2. Non-Conjugated Conductive Radical Polymers

**Scheme 5:**
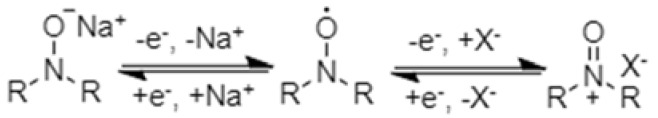
N- and p-doping mechanism of nitroxide radical polymers.

**Figure 8:**
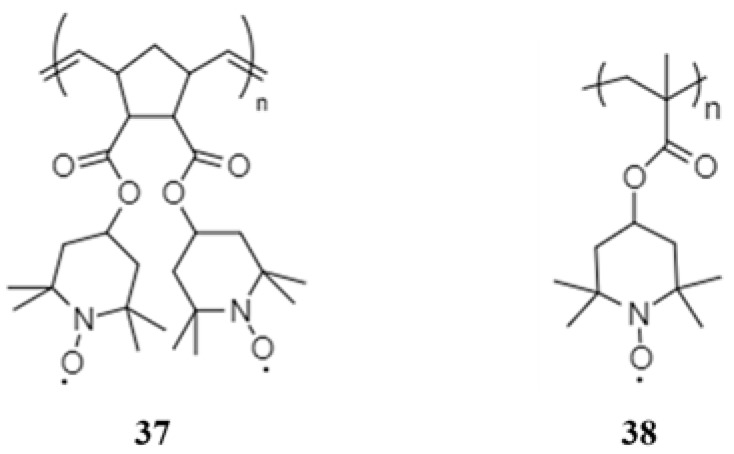
Molecular structures of two non-conjugated conductive radical polymers.

The most widely-explored organic radical polymers for batteries are nitroxide radical polymers (Figure 8). The nitroxide radical polymers can be not only reversibly n-doped to aminoxy anions in cathodic reactions at relatively high voltage but also p-doped to oxoammonium cations in anodic reactions at relative low voltage (Scheme 5).

Nakahara et al. first reported a stable nitroxyl polyradical for rechargeable lithium ion batteries in 2002, and opened a new field of use for plastic batteries [69]. The first application in NIBs reported in 2010 by Dai et al. was a polynorbornene derivative radical polymer, poly[norbornene-2,3-endo, exo-(COO-4-TEMPO)_2_] (**37**) as a cathode active material [70]. The radical polymer **37** delivered an initial discharge capacity of 75 mAh·g^−1^ at a current density of 50 mA·g^−1^ and retained a discharge capacity of 64.5 % at the 50th cycle.

**Figure 9:**
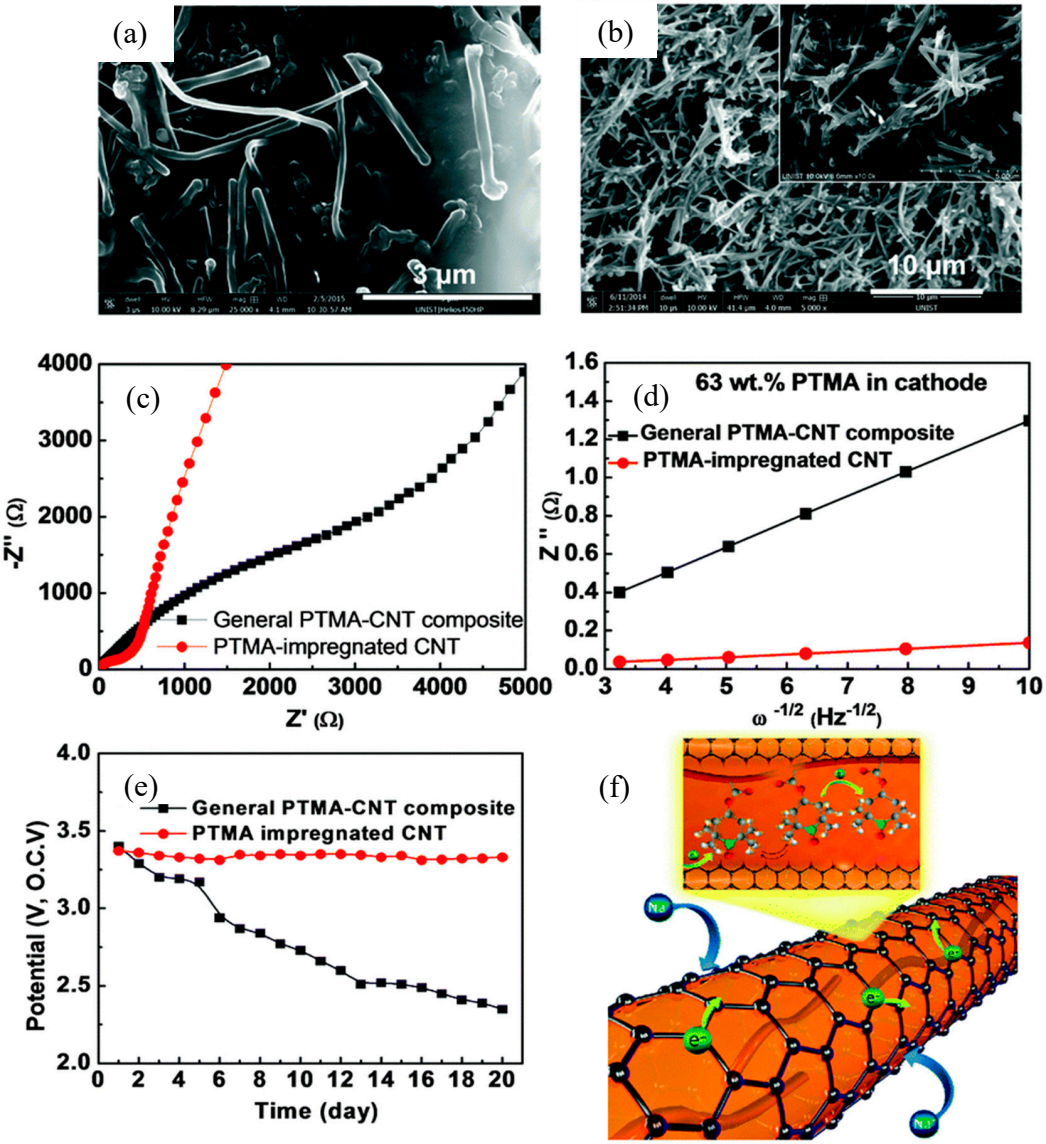
Scanning electronic microscopy images of (**a**) PTMA-CNT composite and (**b**) PTMA-impregnated CNT electrodes; (**c**) impedance spectra and (**d**) the relationship between the imaginary resistance and the square root of frequency in the low-frequency region, (**e**) open circuit voltage during twelve days of PTMA-CNT composite and PTMA-impregnated CNT electrodes as candidate cathode materials for sodium-ion batteries; (**f**) schematic illustration of the PTMA-impregnated CNT structure. Adapted from [71], with permission from © 2008 Royal Society of Chemistry.

Kim et al. designed a unique organic electrode encapsulating the nitroxide radical polymer, poly(2,2,6,6-tetramethylpiperidinyloxy-4-vinylmethacrylate) (PTMA, **38**) into carbon nanotubes (CNT) to form an electrode with high polymer content [71]. To investigate the PTMA-impregnated CNT electrode, the PTMA–CNT composite electrode was compared as a control sample. The non-conducting PTMA layer on the PTMA–CNT composite electrode (Figure 9a) blocked the free passage of ions at the electrode/electrolyte interface, leading to a larger charge-transfer resistance of 3716 Ω (Figure 9c). However, the direct contact between CNTs of the PTMA-impregnated CNT electrode (Figure 9b) improved the electron transfer leading to a decreased resistance of 283 Ω. In addition, the low slope of the relationship between the imaginary resistance and the square root of frequency in the low-frequency region suggested good ion kinetics in the PTMA-impregnated CNT electrode. The self-discharge caused by the dissolution of organic active material can be also minimized effectively by trapping PTMA in CNTs (Figure 9e), leading to improved discharge capacity, cycling performance and rate capability.

The advantages of the radical polymers include fast kinetics due to the small structural change during the oxidation process, stable cell voltage due to the stable structure, and good processability [72]. However, spontaneous self-discharging is one of the biggest challenges for radical polymers due to the dissolution into electrolyte, which functions as a redox shuttle [73]. To overcome this problem, strategies for reducing the dissolution of radical polymers in electrolytes must be developed.

It should be noted that the charge-storage mechanism of the radical polymers involves reversible one-electron redox reaction per repeating unit, and therefore the theoretical capacity of these polymers is strictly limited by the one-electron reaction and the molar mass of the repeat unit. To obtain high-capacity radical polymer electrode materials, it is essential to carefully design the molecular structure of the polymer chain.

## 3. Conclusions

In summary, polymer electrode materials have been shown to exhibit great potential for use in sodium-ion batteries because of the abundance of sodium, structure diversity, composition tenability, and various functional groups. It is believed that polymer batteries represent the future energy storage technology.

While a variety of polymer electrode materials for sodium-ion batteries has been investigated, the research remains in its infancy. More polymeric materials of different structures need to be explored for sodium-ion battery applications. On the basis of our current understanding of sodium-ion storage mechanisms in polymer electrode materials, carbonyl groups, heteroatoms in conjugated system and radicals can be the active sites for interacting with sodium ions. Therefore, it is highly desirable to design new polymers with large density of such active sites.

Most of the polymers are inherently susceptible to dissolve in organic electrolytes, leading to instability against cycling, which must be solved before polymer batteries are commercialized. Salinization appears as a feasible method to improve the stability of the polymer in organic electrolytes. Incorporation of a polymer within an insoluble conductive framework is another route to prevent the dissolution. For example, encapsulation of poly(2,2,6,6-tetramethylpiperidinyloxy-4-vinylmethacrylate) into the carbon nanotubes can not only prevent the dissolution of polymer electrode materials into the electrolyte but also assure the electron transfer by direct contact between carbon nanotubes. Polymers in general have low electron conductivity, causing poor electrochemical performance. Doping with other electronic dopants can be an effective way to improve the electronic conductivity of polymers, which has proved to be efficient for iodine-doped Schiff base polymers with increased conductivity up to the level of semiconductors. Conducting polymers have low capacity, limited by the degree of doping. Copolymerization or incorporation with other high-capacity redox active unit is a useful approach to improve the capacity due to the contribution from the doped redox-active units. Indeed many strategies have been proposed to address issues of polymer electrode materials, but further research is still needed to optimize polymer properties.

The environmental friendliness and multi-functionality of polymers make them essential components in the design of the next generation of organic batteries. The development of rechargeable polymer batteries will certainly attract more and more research interest. It is very likely that emerging new polymer electrode materials with innovative structures and properties will pave the way towards the development of sustainable energy storage devices.

## Figures and Tables

**Table 1:** Comparison of electrode materials for sodium-ion batteries.

Electrode Materials		Advantages	Disadvantages
Inorganic materials		Good conductivity	Potential environmental contamination; Large volume changes; Irreversible phase transitions.
Organic materials	Small organic molecules	Environmental friendliness; Low cost; Structural diversity; Tenability; Flexibility.	High solubility in electrolyte; Poor conductivity.
	Polymers	The same advantages of small organic molecules with lower solubility.	Potential solubility in electrolyte; Poor conductivity except conducting polymers.

**Table 2:** Summary of electrochemical properties of some polymer electrode materials.

No.	Cathodic/Anodic Peak Potential (V vs. Na/Na^+^)	The 1st and 2nd Discharge Capacity(mAh·g^−1^)	Cycling Performance: Discharge Capacity Retention/Cycle/Current Density (mA·g^−1^)	Rate Capability: Discharge Capacity (mAh·g^−1^)/Current Density (mA·g^−1^)	Conductive Additive (wt. %)	Electrolyte *
**1** [37]	1.90/2.20	112, NA	89%/600/25	137.1/50, 38.9/20000	30	1 M NaPF_6_ in EC/DMC (1/1)
**2** [38]	1.86/1.97, 2.45/2.75	126, 121	90%/50/100	120/25, 90/800	30	1 M NaPF_6_ in PC
**3** [39]	1.80/2.10, 2.25/2.4	140, NA	90%/500/140	140/140, 84/2520	50	1 M NaClO_4_ in EC/DEC (1/1)
**4** [40]	2.07/2.22, 2.27/2.47	184, 170	74%/20/50	NA	30	5 M NaNO_3_ in H_2_O
**5** [41]	0.75/1~2, 1.25/1~2	NA	NA	125/25, 43/2000	30	1 M NaClO_4_ in EC/PC (1/1) plus 0.3 wt% FEC
**6** [42]	1.90/2.30, 2.10/2.80	122, NA	70%/100/50	NA	30	Saturated NaPF_6_ in DME/DOL (1/1)
**7** [43]	1.45/1.70, 1.80/2.10, 2.10/2.45	202, NA	95%/150/50	NA	40	Saturated NaPF_6_ in DME/DOL (1/1)
**8** [43]	1.30/1.75, 1.75/2.25	179, NA	92%/150/50	NA	40	Saturated NaPF_6_ in DME/DOL (1/1)
**9** [44]	1.42/1.78, 1.65/2.0, 2.00/2.25, 2.12/2.40	211, NA	57%/150/50	NA	40	Saturated NaPF_6_ in DME/DOL (1/1)
**10** [44]	1.52/1.68, 1.85/2.0, 2.15/2.30	234, NA	82%/150/50	200/50, 65/1000	40	Saturated NaPF_6_ in DME/DOL (1/1)
**11** [44]	1.35/1.65, 1.80/2.0, 1.90/2.25	242, NA	62%/150/50	NA	40	Saturated NaPF_6_ in DME/DOL (1/1)
**12** [44]	1.42/1.58, 1.85/2.15	248, NA	75%/150/50	NA	40	Saturated NaPF_6_ in DME/DOL (1/1)
**13** [45]	1.00/1.30, 1.76/2.20	1161.2, NA	15%/100/500	330.8/100, 102.3/5000	30	1 M NaClO_4_ in EC/DEC (1/1)
**14** [45]	0.90/1.30, 1.40/1.90	NA	NA	137.0/100, 69.4/5000	30	1 M NaClO_4_ in EC/DEC (1/1)
**15** [46]	1.46/1.8, 1.85/2.18	185, NA	100%/200/1600	210/400, 160/6000	50	Saturated NaPF_6_ in DME/DOL (1/1)
**16** [47]	2.08	247, NA	68%/100/50	NA	30	1 M NaN(CF_3_SO_2_)_2_ in DME/DOL (1/1)
**17** [48]	NA	1081, NA	47%/1024/50	433/100, 122/3200	20	1 M NaPF_6_ in EC/DEC (1/1)
**18** [50]	1.02/1.11, 1.38/1.44	309, 224.9	45%/500/500	225/100, 131/1000	30	1 M NaClO_4_ in EC/PC (1/1)
**19** [52]	0.47/0.85	150, NA	NA	NA	20	1M NaN(SO_2_F)_2_ in Me-THF
**20** [52]	0.37/0.79	NA	NA	NA	20	1 M NaN(SO_2_F)_2_ in Me-THF
**21** [52]	0.75/0.95	360, NA	53%/75/26	NA	50	1 M NaN(SO_2_F)_2_ in Me-THF
**22** [52]	0.65/0.79	NA	NA	NA	20	1 M NaN(SO_2_F)_2_ in Me-THF
**23** [52]	0.34/0.87	NA	NA	NA	20	1 M NaN(SO_2_F)_2_ in Me-THF
**24** [52]	0.60/0.87	NA	NA	NA	20	1 M NaN(SO_2_F)_2_ in Me-THF
**25** [53]	0.66, 1.04	206, NA	89%/25/19.7	NA	20	1 M NaN(SO_2_F)_2_ in Me-THF
**28** [46]	3.4/3.7	91, NA	97%/200/500	96/200, 88/2000	50	Saturated NaPF_6_ in DME/DOL (1/1)
**29**	NA	120, NA	94%/20/50	NA	20	5 mol% NaClO_4_ in SCN [57]
	2.65/3.15, 3.5/3.75	181, NA	96%/50/50	180/50, 165/200	20	1M NaPF_6_ in EC/DMC/DEC (1/1/1) [62]
**30** [58]	2.8/3.0, 3.4/3.6	122, NA	97%/200/100	123/100, 76/800	10	1 M NaPF_6_ in EC/DEC (1/1)
**31** [59]	3.3/3.5, 3.6/3.7	103, NA	70%/100/50	92/100, 43/400	40	1M NaPF_6_ in EC/ DEC (1/1)
**32** [60]	3.0/3.3	75, NA	100%/100/40	NA	30	1M NaPF_6_ in EC/ DEC (1/1)
**33** [63]	2.5, 3.7	115, NA	82%/50/50	105/100, 85/800	10	1M NaPF_6_ in EC/ DEC (1/1)
**34** [64]	2.35/2.75	120, NA	92%/100/50	135/50, 75/1600	10	1M NaPF_6_ in EC/ DEC (1/1)
**35**	NA	471.2, NA	40%/100/200	159.2/1800, 137.7/7200	20	1 M NaClO_4_ in PC [65]
	2.5/2.8	70, NA	79%/1000/400	100/20, 69/320	10	1 M NaClO_4_ in EC/PC (1/1) [66]
	2.5/2.7	83, NA	100%/120/300	107/100, 75/300	15	1 M NaPF_6_ in DEGDME [67]
**36** [68]	3.54/3.78	121, NA	79%/10/20	60/100, 10/500	10	1 M NaClO_4_ in PC
**37** [70]	3.38/3.42	75, 73	65%/50/50	NA	60	1M NaClO_4_ in EC/DMC (1/1)
**38** [71]	2.1/2.51, 3.36/3.51	217, NA	92%/100/112.5	222/22.5, 190/1125	30	1M NaClO_4_ in EC/DEC (1/1)

* EC: ethylene carbonate, DMC: dimethyl carbonate, PC: propylene carbonate, DEC: diethyl carbonate, FEC: fluoroethylene carbonate, DME: dimethoxy ethane, DOL: dioxolane, Me-THF: methyltetrahydrofuran, SCN: succinonitrile, DEGDME: diethylene glycol dimethyl ether. 1/1 and 1/1/1: volume ratio.
